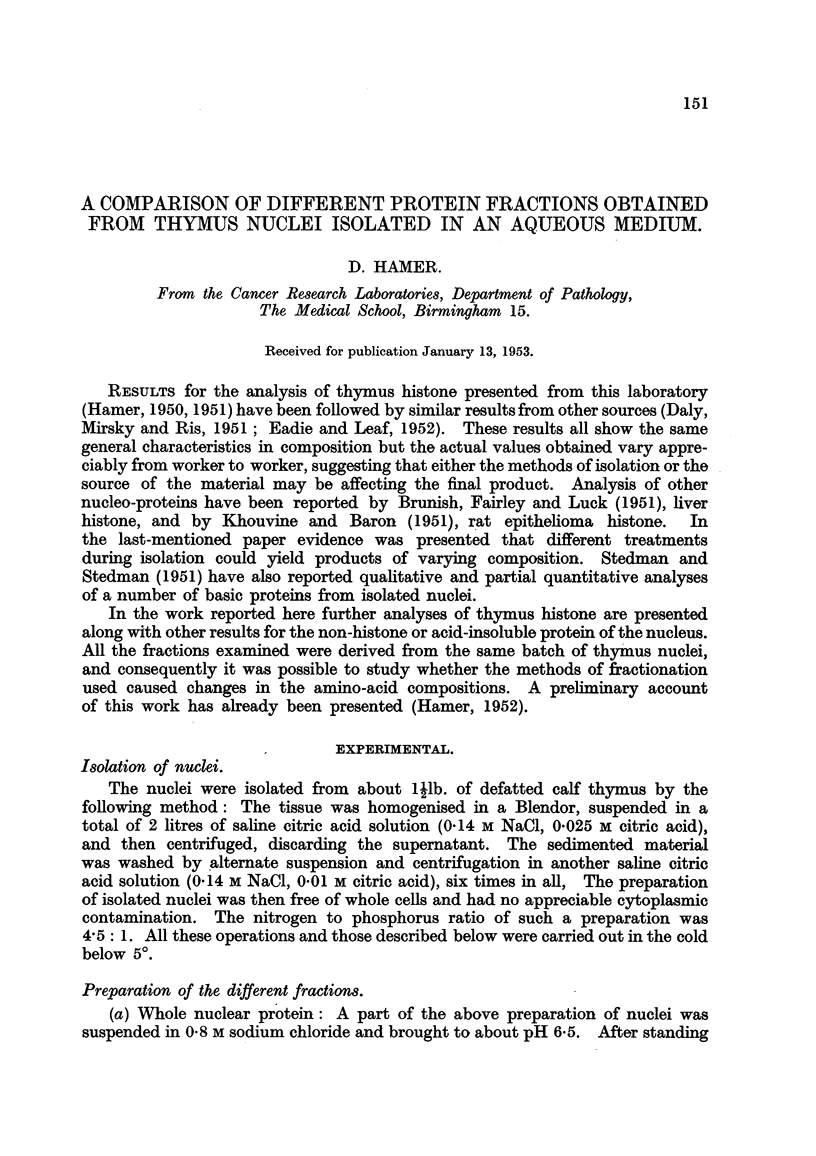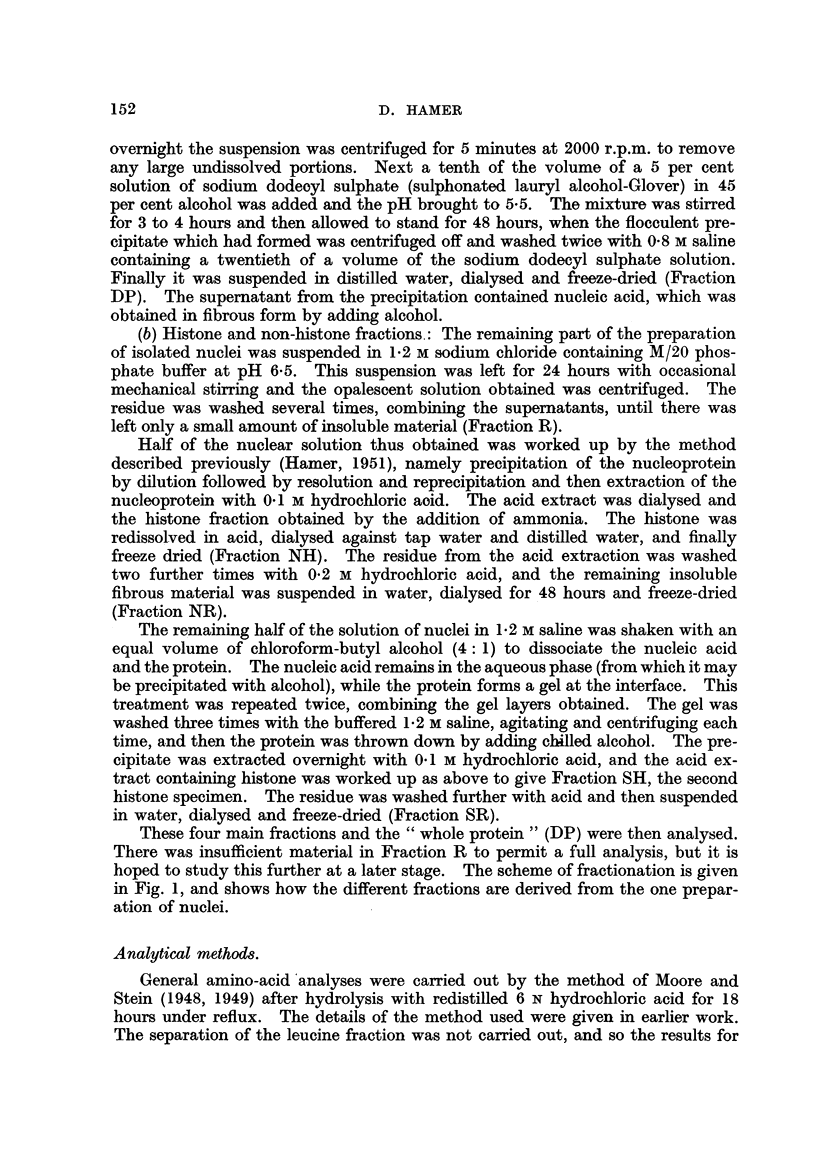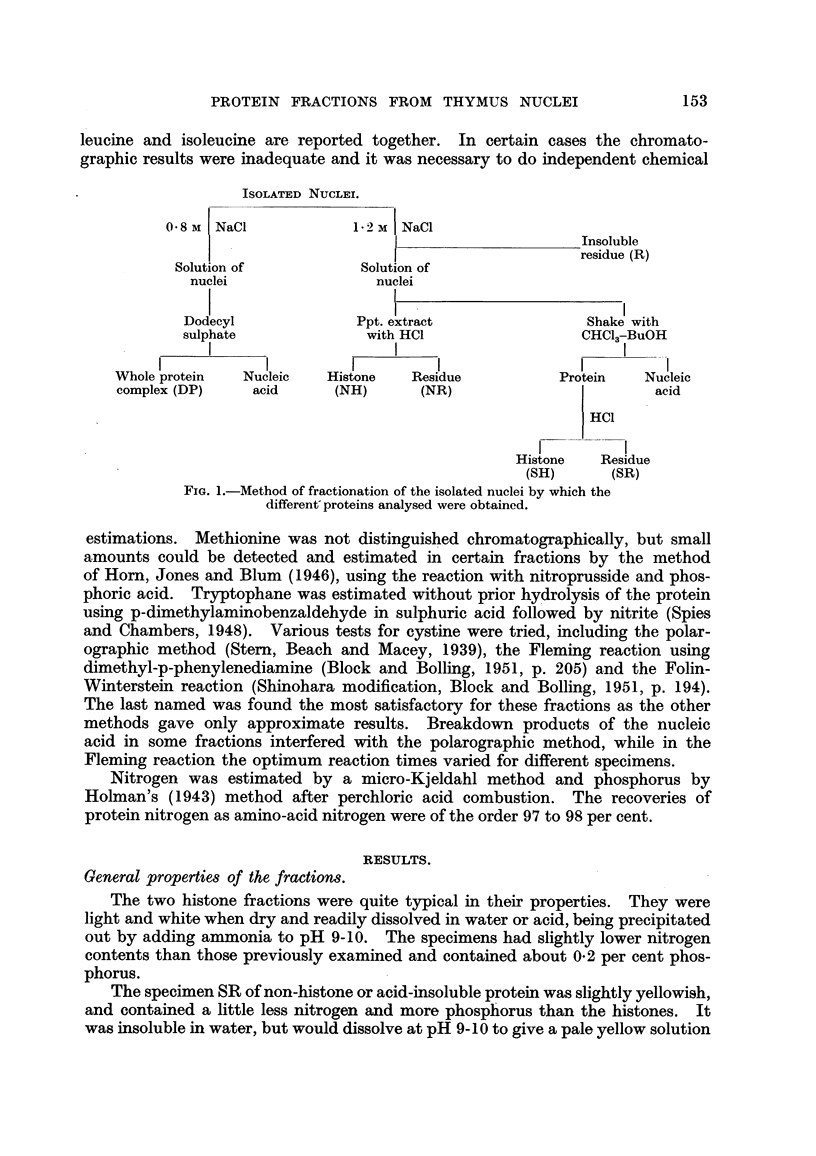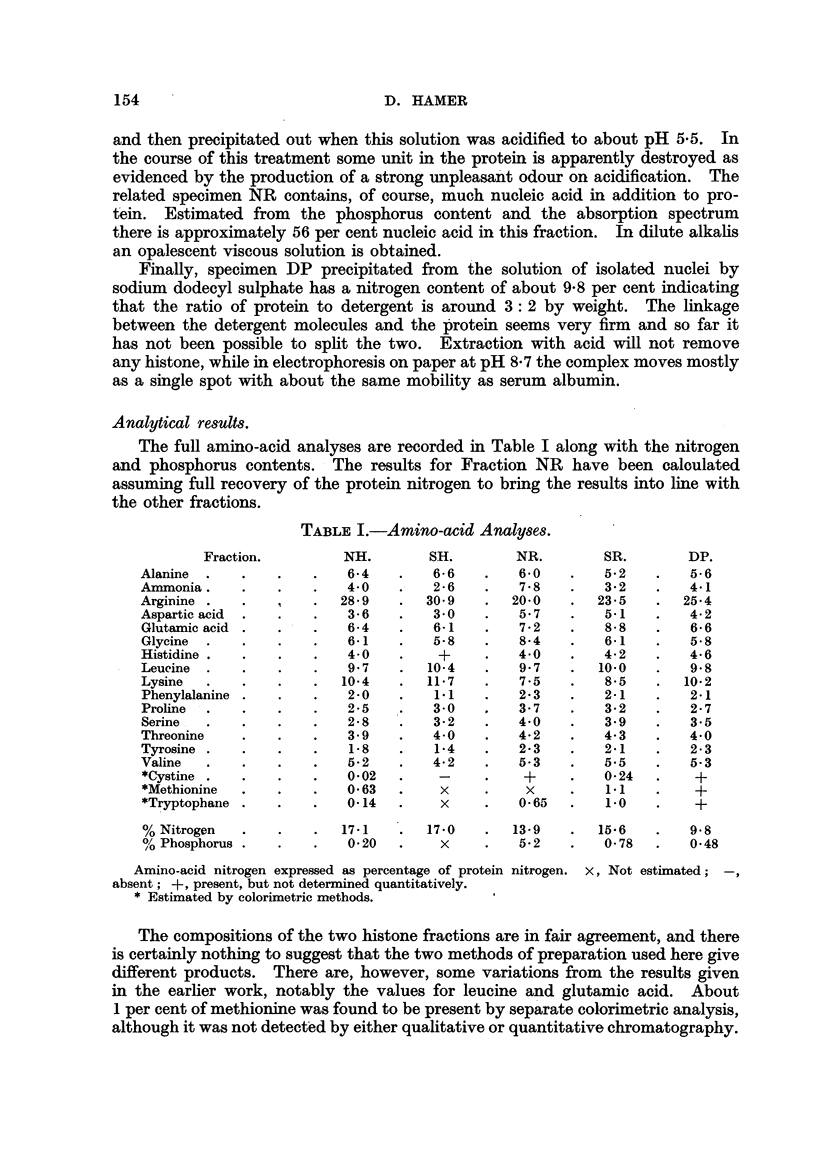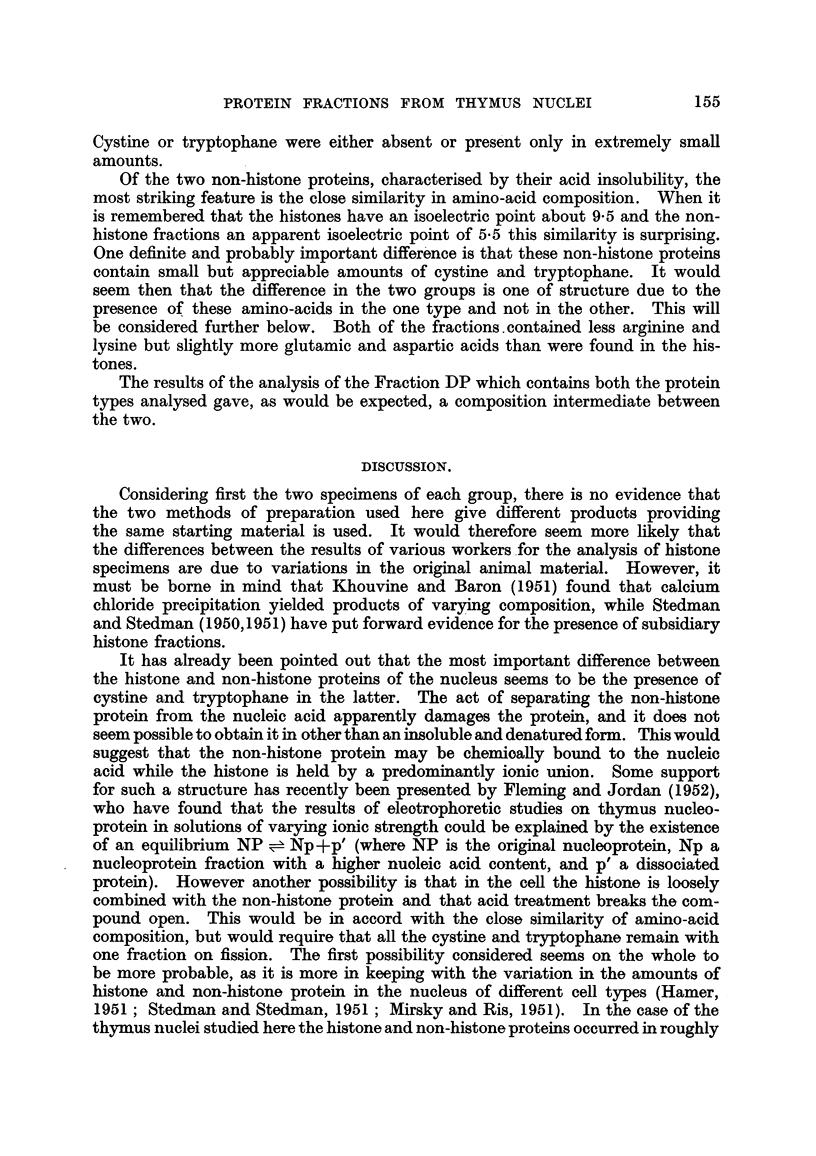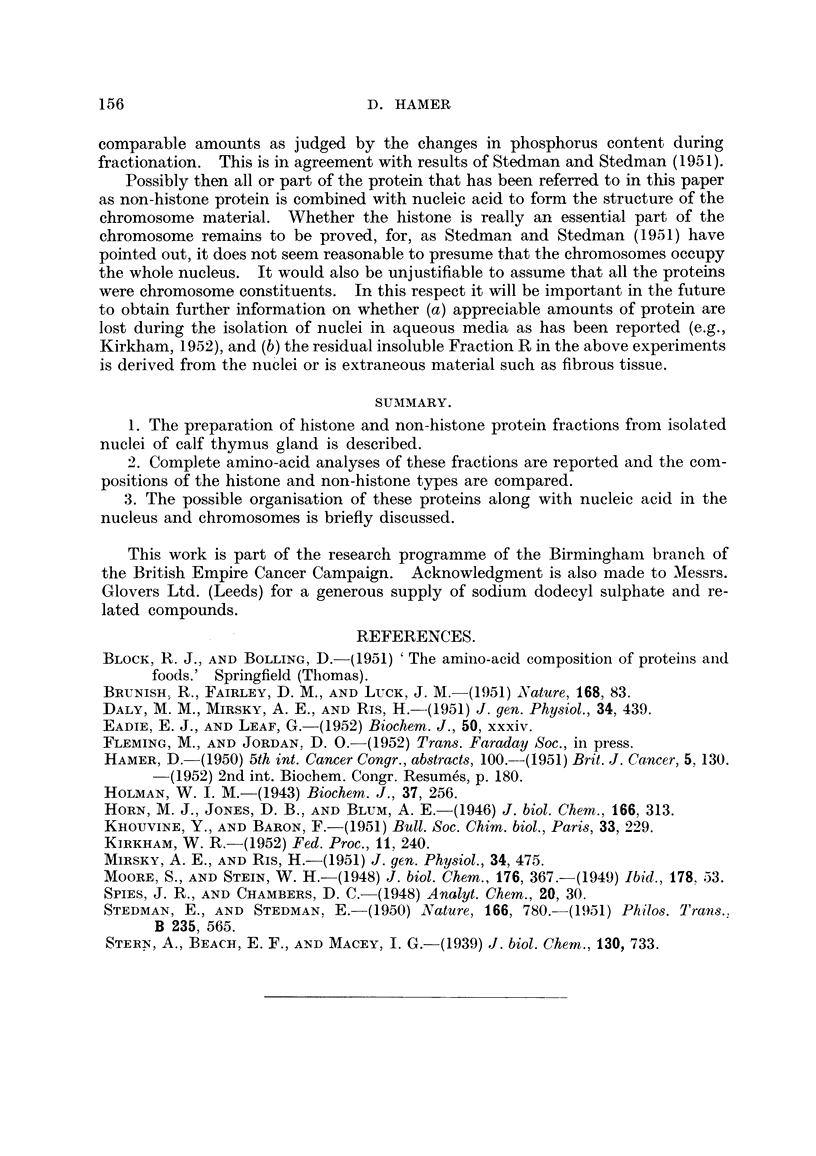# A Comparison of Different Protein Fractions Obtained from Thymus Nuclei Isolated in an Aqueous Medium

**DOI:** 10.1038/bjc.1953.14

**Published:** 1953-03

**Authors:** D. Hamer


### A COMPARISON OF DIFFERENT PROTEIN FRACTIONS OBTAINED FROM THYMUS NUCLEI ISOLATED IN AN AQUEOUS MEDIUM.

D. HAMER.

*From the Cancer Research Laboratories, Department of Pathology, The Medical School, Birmingham* 15.

Received for publication January 13, 1953.

RESULTS for the analysis of thymus histone presented from this laboratory (Hamer, 1950, 1951) have been followed by similar results from other sources (Daly, Mirsky and Ris, 1951 ; Eadie and Leaf, 1952). These results all show the same general characteristics in composition but the actual values obtained vary appreciably from worker to worker, suggesting that either the methods of isolation or the source of the material may be affecting the final product. Analysis of other nucleo-proteins have been reported by Brunish, Fairley and Luck (1951), liver histone, and by Khouvine and Baron (1951), rat epithelioma histone. In the last-mentioned paper evidence was presented that different treatments during isolation could yield products of varying composition. Stedman and Stedman (1951) have also reported qualitative and partial quantitative analyses of a number of basic proteins from isolated nuclei.

In the work reported here further analyses of thymus histone are presented along with other results for the non-histone or acid-insoluble protein of the nucleus. All the fractions examined were derived from the same batch of thymus nuclei, and consequently it was possible to study whether the methods of fractionation used caused changes in the amino-acid compositions. A preliminary account of this work has already been presented (Hamer, 1952).

### EXPERIMENTAL.

*Isolation of nuclei.*

The nuclei were isolated from about 1½lb. of defatted calf thymus by the following method : The tissue was homogenised in a Blendor, suspended in a total of 2 litres of saline citric acid solution (0·14 M NaCl, 0·025 M citric acid), and then centrifuged, discarding the supernatant. The sedimented material was washed by alternate suspension and centrifugation in another saline citric acid solution (0·14 M NaCl, 0·01 M citric acid), six times in all, The preparation of isolated nuclei was then free of whole cells and had no appreciable cytoplasmic contamination. The nitrogen to phosphorus ratio of such a preparation was 4·5 : 1. All these operations and those described below were carried out in the cold below 5°.

*Preparation of the different fractions.*

(*a*) Whole nuclear protein : A part of the above preparation of nuclei was suspended in 0·8 M sodium chloride and brought to about pH 6·5. After standing overnight the suspension was centrifuged for 5 minutes at 2000 r.p.m. to remove any large undissolved portions. Next a tenth of the volume of a 5 per cent solution of sodium dodecyl sulphate (sulphonated lauryl alcohol-Glover) in 45 per cent alcohol was added and the pH brought to 5·5. The mixture was stirred for 3 to 4 hours and then allowed to stand for 48 hours, when the flocculent precipitate which had formed was centrifuged off and washed twice with 0·8 M saline containing a twentieth of a volume of the sodium dodecyl sulphate solution. Finally it was suspended in distilled water, dialysed and freeze-dried (Fraction DP). The supernatant from the precipitation contained nucleic acid, which was obtained in fibrous form by adding alcohol.

(*b*) Histone and non-histone fractions.: The remaining part of the preparation of isolated nuclei was suspended in 1·2 M sodium chloride containing M/20 phosphate buffer at pH 6·5. This suspension was left for 24 hours with occasional mechanical stirring and the opalescent solution obtained was centrifuged. The residue was washed several times, combining the supernatants, until there was left only a small amount of insoluble material (Fraction R).

Half of the nuclear solution thus obtained was worked up by the method described previously (Hamer, 1951), namely precipitation of the nucleoprotein by dilution followed by resolution and reprecipitation and then extraction of the nucleoprotein with 0·1 M hydrochloric acid. The acid extract was dialysed and the histone fraction obtained by the addition of ammonia. The histone was redissolved in acid, dialysed against tap water and distilled water, and finally freeze dried (Fraction NH). The residue from the acid extraction was washed two further times with 0·2 M hydrochloric acid, and the remaining insoluble fibrous material was suspended in water, dialysed for 48 hours and freeze-dried (Fraction NR).

The remaining half of the solution of nuclei in 1·2 M saline was shaken with an equal volume of chloroform-butyl alcohol (4 : 1) to dissociate the nucleic acid and the protein. The nucleic acid remains in the aqueous phase (from which it may be precipitated with alcohol), while the protein forms a gel at the interface. This treatment was repeated twice, combining the gel layers obtained. The gel was washed three times with the buffered 1·2 M saline, agitating and centrifuging each time, and then the protein was thrown down by adding chilled alcohol. The precipitate was extracted overnight with 0·1 M hydrochloric acid, and the acid extract containing histone was worked up as above to give Fraction SH, the second histone specimen. The residue was washed further with acid and then suspended in water, dialysed and freeze-dried (Fraction SR).

These four main fractions and the " whole protein " (DP) were then analysed. There was insufficient material in Fraction R to permit a full analysis, but it is hoped to study this further at a later stage. The scheme of fractionation is given in Fig. 1, and shows how the different fractions are derived from the one preparation of nuclei.

*Analytical methods.*

General amino-acid analyses were carried out by the method of Moore and Stein (1948, 1949) after hydrolysis with redistilled 6 N hydrochloric acid for 18 hours under reflux. The details of the method used were given in earlier work. The separation of the leucine fraction was not carried out, and so the results for leucine and isoleucine are reported together. In certain cases the chromatographic results were inadequate and it was necessary to do independent chemical

FIG. 1.—Method of fractionation of the isolated nuclei by which the different proteins analysed were obtained.

estimations. Methionine was not distinguished chromatographically, but small amounts could be detected and estimated in certain fractions by the method of Horn, Jones and Blum (1946), using the reaction with nitroprusside and phosphoric acid. Tryptophane was estimated without prior hydrolysis of the protein using p-dimethylaminobenzaldehyde in sulphuric acid followed by nitrite (Spies and Chambers, 1948). Various tests for cystine were tried, including the polarographic method (Stern, Beach and Macey, 1939), the Fleming reaction using dimethyl-p-phenylenediamine (Block and Bolling, 1951, p. 205) and the Folin-Winterstein reaction (Shinohara modification, Block and Bolling, 1951, p. 194). The last named was found the most satisfactory for these fractions as the other methods gave only approximate results. Breakdown products of the nucleic acid in some fractions interfered with the polarographic method, while in the Fleming reaction the optimum reaction times varied for different specimens.

Nitrogen was estimated by a micro-Kjeldahl method and phosphorus by Holman's (1943) method after perchloric acid combustion. The recoveries of protein nitrogen as amino-acid nitrogen were of the order 97 to 98 per cent.

### RESULTS.

### *General properties of the fractions.*

The two histone fractions were quite typical in their properties. They were light and white when dry and readily dissolved in water or acid, being precipitated out by adding ammonia to pH 9-10. The specimens had slightly lower nitrogen contents than those previously examined and contained about 0·2 per cent phosphorus.

The specimen SR of non-histone or acid-insoluble protein was slightly yellowish, and contained a little less nitrogen and more phosphorus than the histones. It was insoluble in water, but would dissolve at pH 9-10 to give a pale yellow solution and then precipitated out when this solution was acidified to about pH 5·5. In the course of this treatment some unit in the protein is apparently destroyed as evidenced by the production of a strong unpleasant odour on acidification. The related specimen NR contains, of course, much nucleic acid in addition to protein. Estimated from the phosphorus content and the absorption spectrum there is approximately 56 per cent nucleic acid in this fraction. In dilute alkalis an opalescent viscous solution is obtained.

Finally, specimen DP precipitated from the solution of isolated nuclei by sodium dodecyl sulphate has a nitrogen content of about 9·8 per cent indicating that the ratio of protein to detergent is around 3 : 2 by weight. The linkage between the detergent molecules and the protein seems very firm and so far it has not been possible to split the two. Extraction with acid will not remove any histone, while in electrophoresis on paper at pH 8·7 the complex moves mostly as a single spot with about the same mobility as serum albumin.

*Analytical results.*

The full amino-acid analyses are recorded in Table I along with the nitrogen and phosphorus contents. The results for Fraction NR have been calculated assuming full recovery of the protein nitrogen to bring the results into line with the other fractions.

TABLE I.—*Amino-acid Analyses.*

| Fraction. | NH. | SH. | NR. | SR. | DP. |
|---|---|---|---|---|---|
| Alanine | 6·4 | 6·6 | 6·0 | 5·2 | 5·6 |
| Ammonia | 4·0 | 2·6 | 7·8 | 3·2 | 4·1 |
| Arginine | 28·9 | 30·9 | 20·0 | 23·5 | 25·4 |
| Aspartic acid | 3·6 | 3·0 | 5·7 | 5·1 | 4·2 |
| Glutamic acid | 6·4 | 6·1 | 7·2 | 8·8 | 6·6 |
| Glycine | 6·1 | 5·8 | 8·4 | 6·1 | 5·8 |
| Histidine | 4·0 | + | 4·0 | 4·2 | 4·6 |
| Leucine | 9·7 | 10·4 | 9·7 | 10·0 | 9·8 |
| Lysine | 10·4 | 11·7 | 7·5 | 8·5 | 10·2 |
| Phenylalanine | 2·0 | 1·1 | 2·3 | 2·1 | 2·1 |
| Proline | 2·5 | 3·0 | 3·7 | 3·2 | 2·7 |
| Serine | 2·8 | 3·2 | 4·0 | 3·9 | 3·5 |
| Threonine | 3·9 | 4·0 | 4·2 | 4·3 | 4·0 |
| Tyrosine | 1·8 | 1·4 | 2·3 | 2·1 | 2·3 |
| Valine | 5·2 | 4·2 | 5·3 | 5·5 | 5·3 |
| *Cystine | 0·02 | — | + | 0·24 | + |
| *Methionine | 0·63 | × | × | 1·1 | + |
| *Tryptophane | 0·14 | × | 0·65 | 1·0 | + |
| % Nitrogen | 17·1 | 17·0 | 13·9 | 15·6 | 9·8 |
| % Phosphorus | 0·20 | × | 5·2 | 0·78 | 0·48 |

Amino-acid nitrogen expressed as percentage of protein nitrogen. ×, Not estimated; —, absent; +, present, but not determined quantitatively.
* Estimated by colorimetric methods.

The compositions of the two histone fractions are in fair agreement, and there is certainly nothing to suggest that the two methods of preparation used here give different products. There are, however, some variations from the results given in the earlier work, notably the values for leucine and glutamic acid. About 1 per cent of methionine was found to be present by separate colorimetric analysis, although it was not detected by either qualitative or quantitative chromatography.

Cystine or tryptophane were either absent or present only in extremely small amounts.

Of the two non-histone proteins, characterised by their acid insolubility, the most striking feature is the close similarity in amino-acid composition. When it is remembered that the histones have an isoelectric point about 9·5 and the non-histone fractions an apparent isoelectric point of 5·5 this similarity is surprising. One definite and probably important difference is that these non-histone proteins contain small but appreciable amounts of cystine and tryptophane. It would seem then that the difference in the two groups is one of structure due to the presence of these amino-acids in the one type and not in the other. This will be considered further below. Both of the fractions contained less arginine and lysine but slightly more glutamic and aspartic acids than were found in the histones.

The results of the analysis of the Fraction DP which contains both the protein types analysed gave, as would be expected, a composition intermediate between the two.

### DISCUSSION.

Considering first the two specimens of each group, there is no evidence that the two methods of preparation used here give different products providing the same starting material is used. It would therefore seem more likely that the differences between the results of various workers for the analysis of histone specimens are due to variations in the original animal material. However, it must be borne in mind that Khouvine and Baron (1951) found that calcium chloride precipitation yielded products of varying composition, while Stedman and Stedman (1950,1951) have put forward evidence for the presence of subsidiary histone fractions.

It has already been pointed out that the most important difference between the histone and non-histone proteins of the nucleus seems to be the presence of cystine and tryptophane in the latter. The act of separating the non-histone protein from the nucleic acid apparently damages the protein, and it does not seem possible to obtain it in other than an insoluble and denatured form. This would suggest that the non-histone protein may be chemically bound to the nucleic acid while the histone is held by a predominantly ionic union. Some support for such a structure has recently been presented by Fleming and Jordan (1952), who have found that the results of electrophoretic studies on thymus nucleoprotein in solutions of varying ionic strength could be explained by the existence of an equilibrium $NP \rightleftharpoons Np + p'$ (where NP is the original nucleoprotein, Np a nucleoprotein fraction with a higher nucleic acid content, and p′ a dissociated protein). However another possibility is that in the cell the histone is loosely combined with the non-histone protein and that acid treatment breaks the compound open. This would be in accord with the close similarity of amino-acid composition, but would require that all the cystine and tryptophane remain with one fraction on fission. The first possibility considered seems on the whole to be more probable, as it is more in keeping with the variation in the amounts of histone and non-histone protein in the nucleus of different cell types (Hamer, 1951 ; Stedman and Stedman, 1951 ; Mirsky and Ris, 1951). In the case of the thymus nuclei studied here the histone and non-histone proteins occurred in roughly comparable amounts as judged by the changes in phosphorus content during fractionation. This is in agreement with results of Stedman and Stedman (1951).

Possibly then all or part of the protein that has been referred to in this paper as non-histone protein is combined with nucleic acid to form the structure of the chromosome material. Whether the histone is really an essential part of the chromosome remains to be proved, for, as Stedman and Stedman (1951) have pointed out, it does not seem reasonable to presume that the chromosomes occupy the whole nucleus. It would also be unjustifiable to assume that all the proteins were chromosome constituents. In this respect it will be important in the future to obtain further information on whether (*a*) appreciable amounts of protein are lost during the isolation of nuclei in aqueous media as has been reported (e.g., Kirkham, 1952), and (*b*) the residual insoluble Fraction R in the above experiments is derived from the nuclei or is extraneous material such as fibrous tissue.

### SUMMARY.

1. The preparation of histone and non-histone protein fractions from isolated nuclei of calf thymus gland is described.

2. Complete amino-acid analyses of these fractions are reported and the compositions of the histone and non-histone types are compared.

3. The possible organisation of these proteins along with nucleic acid in the nucleus and chromosomes is briefly discussed.

This work is part of the research programme of the Birmingham branch of the British Empire Cancer Campaign. Acknowledgment is also made to Messrs. Glovers Ltd. (Leeds) for a generous supply of sodium dodecyl sulphate and related compounds.

### REFERENCES.

Block, R. J., and Bolling, D.—(1951) 'The amino-acid composition of proteins and foods.' Springfield (Thomas).
Brunish, R., Fairley, D. M., and Luck, J. M.—(1951) *Nature*, **168**, 83.
Daly, M. M., Mirsky, A. E., and Ris, H.—(1951) *J. gen. Physiol.*, **34**, 439.
Eadie, E. J., and Leaf, G.—(1952) *Biochem. J.*, **50**, xxxiv.
Fleming, M., and Jordan, D. O.—(1952) *Trans. Faraday Soc.*, in press.
Hamer, D.—(1950) *5th int. Cancer Congr., abstracts*, 100.—(1951) *Brit. J. Cancer*, **5**, 130. —(1952) 2nd int. Biochem. Congr. Resumés, p. 180.
Holman, W. I. M.—(1943) *Biochem. J.*, **37**, 256.
Horn, M. J., Jones, D. B., and Blum, A. E.—(1946) *J. biol. Chem.*, **166**, 313.
Khouvine, Y., and Baron, F.—(1951) *Bull. Soc. Chim. biol., Paris*, **33**, 229.
Kirkham, W. R.—(1952) *Fed. Proc.*, **11**, 240.
Mirsky, A. E., and Ris, H.—(1951) *J. gen. Physiol.*, **34**, 475.
Moore, S., and Stein, W. H.—(1948) *J. biol. Chem.*, **176**, 367.—(1949) *Ibid.*, **178**, 53.
Spies, J. R., and Chambers, D. C.—(1948) *Analyt. Chem.*, **20**, 30.
Stedman, E., and Stedman, E.—(1950) *Nature*, **166**, 780.—(1951) *Philos. Trans.*, **B 235**, 565.
Stern, A., Beach, E. F., and Macey, I. G.—(1939) *J. biol. Chem.*, **130**, 733.